# Leopard-like retinopathy and severe early-onset portal hypertension expand the phenotype of *KARS1*-related syndrome: a case report

**DOI:** 10.1186/s12920-020-00863-1

**Published:** 2021-01-21

**Authors:** Francesca Peluso, Viviana Palazzo, Giuseppe Indolfi, Francesco Mari, Roberta Pasqualetti, Elena Procopio, Claudia Nesti, Renzo Guerrini, Filippo Santorelli, Sabrina Giglio

**Affiliations:** 1 Molecular Medicine, IRCCS Fondazione Stella Maris, Pisa, Italy; 2 Medical Genetics Unit, Meyer Children’s University Hospital, Florence, Italy; 3 Paediatric and Liver Unit, Meyer Children’s University Hospital, Florence, Italy; 4 Paediatric Neurology, Neurogenetics and Neurobiology Unit and Laboratories, Meyer Children’s University Hospital, Florence, Italy; 5 Paediatric Ophthalmology Unit, Meyer Children’s University Hospital, Florence, Italy; 6 Metabolic and Muscular Unit, Meyer Children’s University Hospital of Florence, Florence, Italy; 7 Sabrina Giglio MD, PhD Unit of Medical Genetics, Department of Medical Sciences and Public Health, University of Cagliari, Cagliari, Italy

**Keywords:** *KARS*, Mitochondrial diseases, Encephalohepatopathy, Leopard-like retinopathy, ARSs, Case report

## Abstract

**Background:**

Mutations in lysyl-tRNA synthetase (*KARS1*), an enzyme that charges tRNA with the amino acid lysine in both the cytoplasm and mitochondria, have been associated thus far with autosomal recessive Charcot–Marie–Tooth type CMTRIB, hearing loss type DFNB89, and mitochondrial encephalohepatopathy (MEH) featuring neurodevelopmental disorders with microcephaly, white matter changes, and cardiac and hepatic failure in less than 30 patients.

**Case presentation:**

We report the clinical, biochemical and molecular findings of a 14-month-old girl with severe MEH compatible clinical features, profound sensorineural hearing loss, leopard spot retinopathy, pancytopenia, and advanced liver disease with portal hypertension leading to death at the age of 30 months.

**Conclusions:**

Whole exome sequencing identified two rare variants in *KARS1* gene. Our report expands the allelic and clinical features of tRNA synthase disorders. Moreover, with our report we confirm the usefulness of WES as first tier diagnostic method in infants with complex multisystem phenotypes.

## Background

Aminoacyl tRNA synthetase (ARS) proteins are fundamentally known as the first enzymes of translation that catalyze amino acid attachment to their cognate tRNA. This catalytic process, called tRNA charging, is necessary for the translation of genetic sequences into polypeptide chains [1]. The *KARS1* gene codes for both the mitochondrial and cytoplasmic isoforms of the t-RNA synthase of Lysine, a ubiquitous enzyme responsible for the link between the amino acid Lysine and the cognate RNA transfer [2]. To date, sixteen mutations in the *KARS1* gene have been associated with autosomal recessive Charcot–Marie–Tooth type CMTRIB [3], hearing loss type DFNB89 [4], and mitochondrial encephalohepatopathy (MEH) featuring neurodevelopmental disorders with microcephaly, white matter changes, and cardiac and hepatic failure in 26 patients.

We report the clinical, biochemical, neuroradiological and molecular findings of an additional child. The clinical features observed largely recall the wide spectrum seen in previously reported patients harboring bi-allelic variants in *KARS1* and satisfy the clinical criteria for mitochondrial encephalocardiohepatopathy [5] with limited muscular involvement of oxidative-phosphorylation. Ocular and hepatic findings which have never described up to now will be described.

## Case presentation

**Fig. 1:**
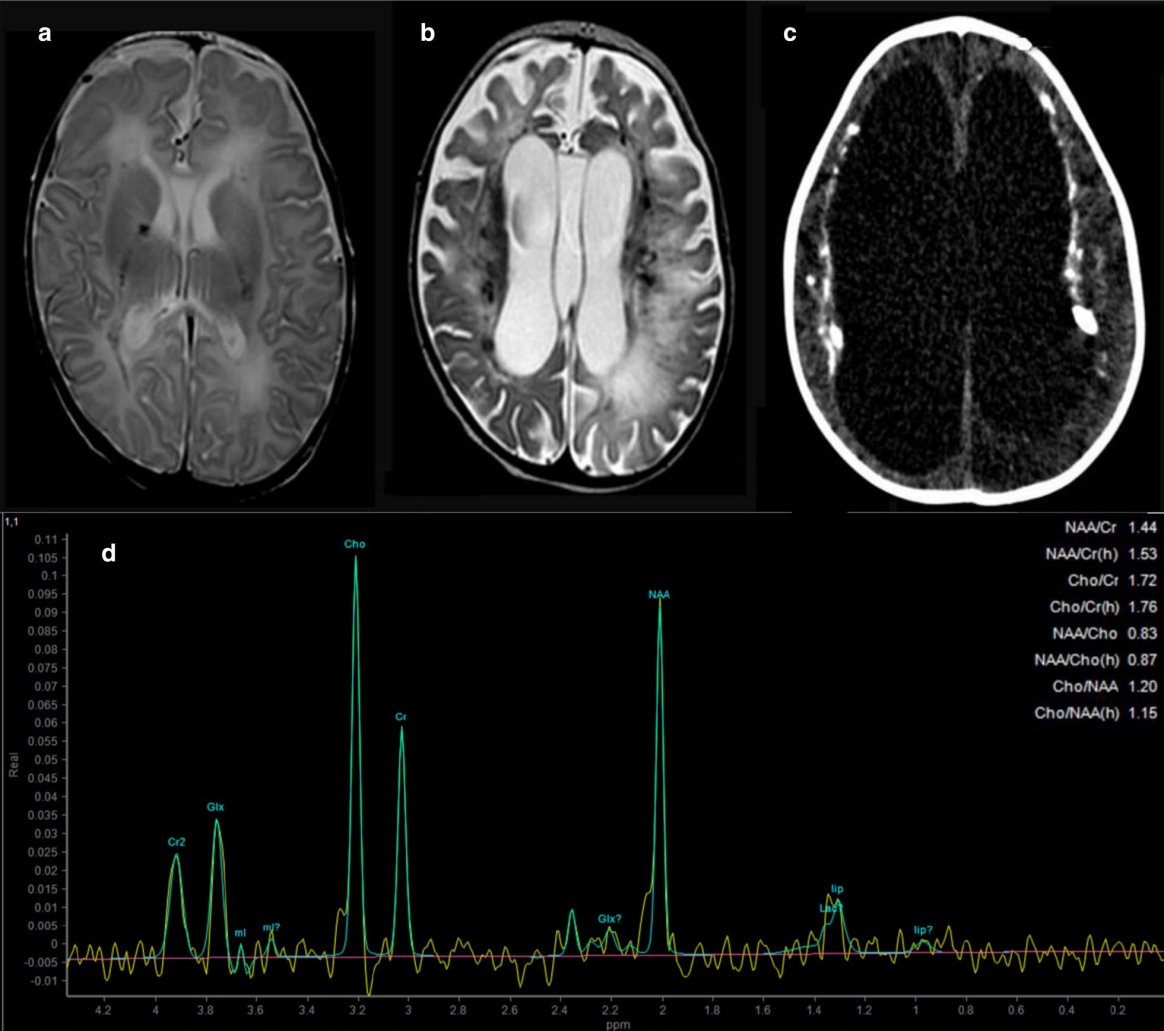
Neuroradiological findings. **a** 1.5T Brain MRI at 4 months (T2-weighted axial section): diffuse and bilateral hyperintense alterations in nucleocapsular and peritrigonal regions, mild calcifications and thin corpus callosum; **b** 1.5T Brain MRI at 30 months (T2-weighted axial section): evolution of progressive damage involving diffusively the white matter leading to cortical, subcortical and cerebellar atrophy with more prominent amount of bilateral calcifications; **c** Computed Tomography (CT) scan at 30 months: generalized and massive cortical and subcortical atrophy with hydrocephalus; **d** MR-Spectroscopy (MRS) at 4 months, volume of interest in left parietal white matter region: reduced NAA/Cr ratio and Cho/Cr ratio and occurrence of a high lactate peak (at 1.3 ppm)

This female child was the first baby born at 38 weeks of gestational age to healthy, unrelated parents from a complicated pregnancy due to intrauterine growth retardation detected at 32 weeks of gestational age. At birth weight was 2240gr (− 1.97 SD), length 48 cm (− 0.39 SD), and cranial circumference 31.5 cm (− 2 SD). First neonatal neurological examination highlighted severe global hypotonia, poor crying and sucking with feeding difficulties. At the age of 4 months, a first brain MRI showed white matter lesions and a major amount of periventricular calcifications. Moreover, MR-Spectroscopy with the voxel localized on left parietal white matter region showed reduced NAA/Cr ratio (1.47) and Cho/Cr ratio (1.40) and an elevated lactate peak (Fig. 1). Follow-up neuroimaging investigations showed progression of cortical and subcortical atrophy and increase of calcifications in subcortical regions and deep brain and the cerebellum.

Neurological and physical examinations at 21 months showed apostural tetraparesis, severe developmental and growth delay, epileptic encephalopathy with spasms, myoclonic and focal seizures. She also showed hyposomatism with low weight 8900 gr (− 2.35 SD) and length 76 cm (− 1.79 SD), microcephaly with reduced head circumference of 39 cm (− 5 SD) at 14 months, and bilateral neurosensorial hearing loss. Echocardiogram revealed pulmonary valve stenosis.

**Fig. 2:**
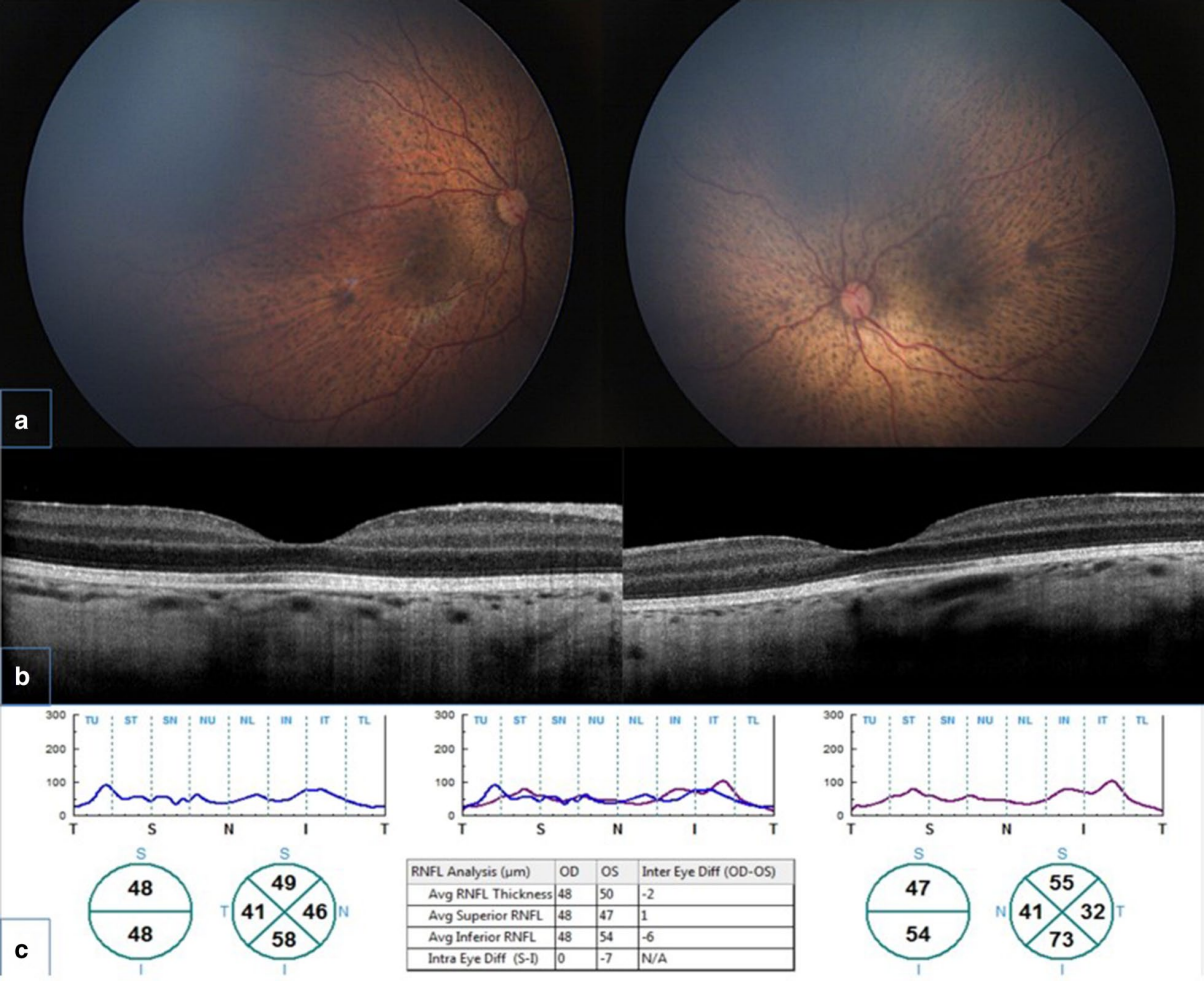
SD-OCT scan findings. **a** Wide field retinal imaging showing ophthalmoscopic appearance of bilateral leopard spot retinopathy and mild hypoplasic appearance of optic nerve head; **b** SD-OCT scan showing normal foveal profile and retinal segmentation; **c** SD-OCT optic nerve scan showing severe and bilateral pRNFL thinning coherent with a neurodegenerative process and bilateral optic atrophy

Ophthalmological examination showed exotropia and poor fixation. Indirect fundus examination revealed the presence of a bilateral, hypoplasic appearance of the optic nerve head and bilateral, diffuse and mottled retinal pigmentation described as “leopard spot” retinopathy. A first spectral domain optical coherence tomography (SD-OCT) scan at age of 3 months revealed subnormal peripapillary retinal nerve fiber layer (pRNFL) in both eyes with overall preserved retinal architecture. A second SD-OCT scan performed at 14 months of age revealed a severe thinning of pRNFL and retinal thickness in both eyes suggestive of progressive retinal degeneration (Fig. 2).

Liver ultrasound at 5 months of age showed a nodular liver surface, round edge, hypoechoic nodules in the liver parenchyma and calcifications in the 6th hepatic segment. The portal vein was non-obstructed, and the spleen was normal (length 4.7 cm). Serum aminotransferases, gamma-glutamil-transpeptidase, albumin and international normalized ratio were normal. Total serum bile acids were slightly elevated (2 times the upper limit of normal). She was occasionally found to have portal hypertension at 5 months of age (grade 2 esophageal varices) and at 14 months of age she presented with acute hematemesis from esophageal varices and then started on propranolol (1–2 mg/kg/day). The child underwent a complete diagnostic work-up and the most common causes of cholestasis were excluded.

High levels of serum lactate (4.9 mM/L, reference range 0.63–2.44 mM/L) and normal pyruvate value were also detected.

**Fig. 3:**
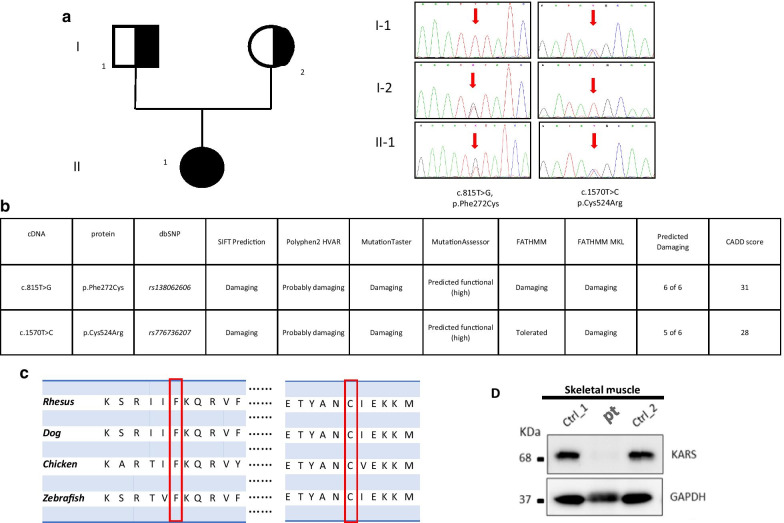
Molecular findings in this patient. **a** Pedigree and electropherograms; **b** in silico predictions using multiple prediction software programs; **c** multiple species alignment; **d** Western Blot analysis in muscle biopsy homogenate

Shortly after, clinical manifestations evolved to a progressive pancytopenia with the observation of ring sideroblasts at a bone marrow biopsy suggestive of a Pearson marrow-pancreas-like syndrome in spite of normal metabolic investigations in blood and urine. Also, the minimal alterations of the respiratory chain complex enzyme activities in a skeletal muscle biopsy and lack of deletion/duplication or punctuate mutations in the mitochondrial genome did not support this possibility. The clinical situation of the baby worsens, and she died at 30 months of life. Array-CGH analysis was negative. Whole exome sequencing (WES) in the family trio (Additional file 1) revealed the paternal c.815T>G/p.[Phe272Cys] (*rs138062606*) and the maternal c.1570T>C/p.[Cys524Arg] (*rs776736207*) (Fig. 3a) variants in *KARS1* (NM 001130089.1). Paternal mutation has already reported elsewhere [6], while the other variant has not yet been described. Both mutations are ultra-rare and in silico predictions using multiple prediction software programs indicate that the two variants are probably pathogenetic (Fig. 3b) and both alter highly conserved nucleotides in the various species (Fig. 3c). Western Blot (WB) analysis in muscle biopsy homogenate using a rabbit polyclonal anti-*KARS* antibody (Proteintech; dilution 1:500) showed a severe reduction of the gene product (Fig. 3d, Additional files 2, 3, 4). This implies an impairment of mitochondrial protein synthesis machinery, though this was not directly tested.

## Discussion and conclusions

Whole exome sequencing allows us to identify two rare genetic variants and functional studies (WB in muscle biopsy) showed a severe reduction of the gene product supporting the direct role of the *KARS1* gene in this child’s disease.

The clinical features observed in our patient largely recall the wide spectrum seen in the 26 reported patients harboring bi-allelic variants in *KARS1* (Table 1) and satisfy the clinical criteria for mitochondrial encephalocardiohepatopathy with limited muscular involvement of oxidative phosphorylation [5].

**Table 1:** Previous reported patients in scientific literature

Patients	Age of onset	Age of follow up	Sex	Cerebral atrophy	Cerebellar atrophy	Cerebral calcification	Location of cerebral calcifications	Cerebellar calcification	Location of cerebellar calcifications
1	6 m	3y (Ϯ)	M						
2	6w	10y	M						
3	5w	5y	F		Loss of subcortical WM volume				
4	72d	18 m	M						
5	9 m		M						
6	18 m	14y	F						
7	1y	26y	F						
8	1y	21y	M						
9	1 m	18y	F			Y(2y)			
10	1 m	15y	F						
11	3 m	19 m	M			Y	Thalami, anterior commissure, anterior arms of the internal capsules, along the opticall pathways, midbrain	Y	Cerebellar dentate nuclei
12	6 m	7y	M	Y(3y)	Y(7Y)	Y(3y)	Periventricular WM, paons, thalami, internal capsules and calcarine cortex	Y	Cerebellar WM
13	6 m	18 m	F			y(6 m)		Y	Internal capsules, deep and periventricular WM
14	28y	33y (Ϯ)	F						
15	Birth	12y (Ϯ)	M			Y(nascita at birth)	WM		
16	Birth	2y (Ϯ)	M	Low density of WM		y(2y)			
17	Birth	4y (Ϯ)	M			y(3y)	Internal capsule		
18	Birth	8y (Ϯ)	M	Low density of WM		y(nascita at birth)			
19	Birth	12y	M	Low density of WM		y(nascita at birth)			
20	Birth	12y (Ϯ)	M	Low density of WM		y(2y)	Internal capsule		
21	Birth	3y	M						
22	25y	27y (Ϯ)	F						
23	15y	35y	M						
24	35y	32y	M						
25	Birth	3y	M					Y	Cerebellar nuclei
26	Birth	12y (Ϯ)	F		Y (cerebellar white loss)	Y	Basal ganglia, frontal, parietal lobes		
27	Birth	30 m (Ϯ)	F	Y	Y	Y	Semioval center, corona radiata, periventricular WM, thalamus, brainstem	Y	Dentate nucleus

Patients	Spinal calcification	Location of spinal calcifications	WM abnormalities	MRI spectroscopy	Repiratory chain	Histological muscle biopsy	Plasmatic organic acids and aminoacids	Cerebrospinal fluid biogenic amines	Liver	Deafness
1					Increased mtDNA in muscle		↑lactate and alanine			Y
2			Deep WM							
3										
4										
5					I, IV		↑lactate			
6					IV, I + IV					
7			Frontal WM, corpus callosum							Y
8			Periventricular WM, corpus callosum							Y
9										Y
10										Y
11	Y	Tracl-like calcifications along the medullary pathways	U-fibers, internal and external capsules, brainstem, cerebellum	↑lactate and lipids, ↑choline/creatine, ↑myoinositol/creatine, ↓*N*-acetylaspartate/creatine	I, II, I + III, II + III	normal	Moderate ketosis,↑ alanin	Biopterin		Y
12	Y	C6-T1	Deep cerebellum WM, middle cerebellar peduncles, brainstem, U-fiber, posterior arm of the internal and external capsules, thalami	↓*N*-acetylaspartate, ↑ lactate			↑lactate and pyruvate		Hepatomegaly, ↑ transaminase	Y(profound)
13	Y(12 m)	Dentate nuclei	Centrum semiovale, corona radiata, posterior arm of the internal and external capsules, thalami, cerebellar amd deep WM and brainstem, V cranial nerves, cervical and dorsal columns				↑lactate and pyruvate		↑ transaminase, vascular disturbance with nodular regenerative hyperplasia	Y
14			Dentate nucleus, optic radiations, corpus callosum splenium		I,IV	COX-neg fibers	↑lactate			Y
15			Posterior limbs of the internal capsules, thalami							Y
16	Y(2y)									Y
17			Precentral gyri	↑lactate						Y
18	Y						↑lactate and pyruvate			Y
19	Y						↑lactate and pyruvate			Y
20			Internal capsules	↑lactate in cerebral WM						Y
21	Y (and atrophy)									Y
22			Inner capsule							Y
23			Periventricular WM							Y
24										Y
25									Abnormal ultrasound	Y
26			Deep WM of both cerebral hemispheres, corticospinal tracts				↑lactate	↑proteins		Y
27				↓NAA/Cr ratio, ↓ Cho/Cr ratio, presence of lactate	I + III, IV		↑lactate and glycine		Cirrhotic hepatopathy, polylobulated liver aspect, presence of bleeding esophageal varices, liver calcifications	Y

Patients	CNS	Microcephaly	Hemato	Seizures	Ocular	Scoliosis	Heart	Others	Allele1	Allele2	Author
1	Development delay, hypotonia, dystonia				Strabism, ophtalmoplegia				c.1760C>T p.Thr587Met	c.683C>T p.Pro228Leu	Lieber (2013) Neurology 80, 1762
2		Y		Y	Nystagmus, delayed PEV				c.1396C>T p.Arg466Trp	c.1657G>A p.Glu553Lys	McMillan (2015) J Child Neurol 30, 1037
3									c.1396C>T p.Arg466Trp	c.1657G>A p.Glu553Lys	McMillan (2015) J Child Neurol 30, 1037
4	Hypertonicity, extreme irritability, psychomotor delay	Y		Y (West sindrome)	Nystagmus, abnomal ocular movements			Failure to thrive	c.169G>C p.Ala57Pro	chr16: 75,672,800–75,680,400 loss of starting codon	Joshi C. (2016) Biomed Res Int. 2016:6,421,039
5	Development delay			Y	Nystagmus		Hypertrophic cardiomyopathy		c.1427T>A p.Val476Asp	c.1037T>C p.Ile346Thr	Kohda (2016) PLoS Genet 12, e1005679
6	Mild psychomotor delay, intellectual disability, mild myopathy						Hypertrophic cardiomyopathy		c.1133T>A p.Leu378Hys	c.1253C>G p.Pro418Arg	Verrigni (2017) Clin Genet 91, 918
7	Progressiv ecognitive impairment								c.1514G>A p.Arg505Hys	c.1597C>T p.Pro533Ser	Zhou (2017) Hum Mutat 38, 1740
8	Progressiv ecognitive impairment								c.1514G>A p.Arg505Hys	c.1597C>T p.Pro533Ser	Zhou (2017) Hum Mutat 38, 1740
9	Development delay, hypotonia	Y		Y					c.1577C>T p.Ala526Val	c.1466T>G p.Phe489Cys	Murray (2017) J Pediatr Genet 6, 77
10	Development delay	Y		Y					c.1577C>T p.Ala526Val	c.1466T>G p.Phe489Cys	Murray (2017) J Pediatr Genet 6, 77
11	Severe spastic tetraplegia, progressive hypertonus, absense of voluntary movementes	Y		Hypertonic seizures	VEP and ERG mild conduction delay				c.1514G>A p.Arg505His	c.1514G>A p.Arg505His	Ardissone A (2018) Orphanet J Rare Dis 13(1):45
12	Absence of spontaneus movements and postural control, spastic tetraparesis with extrapyramidal signs, absence of language		Microcytic hypocromic anemia		Nystagmus, bilateral optic atrophy	Marked			c.1124A>G p.Tyr375Cys	c.381C>G p.Phe127Leu	Ardissone A (2018) Orphanet J Rare Dis 13(1):45
13	Spastic tetraplegia, absent postural control, poor spontaneus movements	Y	Microcytic hypocromic anemia		Delayed VEP, mild retinal depigmentation				c.815T>G p.Phe272Cys	c.1043G>A p.Arg348His	Ardissone (2018) Orphanet J Rare Dis 13(1):45
14	Cerebellar ataxia				Concentric decrease of peripheral isopter				c.683C>T p.Pro228Leu	c.871T>G p.Phe291Val	Scheidecker (2019) Hum Mutat 40, 1826
15	Hypotonia, difficulty feeding, delayed development			Y	Nystagmus				c.1786C>T p.Leu596Phe	c.1786C>T p.Leu596Phe	Itoh (2019) Brain 142(3):560–573
16	Hypotonia, severe development delay		Anemia, thrombocytopenia,	Y	Nystagmus			Distal renal tubular acidosis	c.1786C>T p.Leu596Phe	c.879 + 1G>A p.Glu252_Glu293del	Itoh (2019) Brain 142(3):560–573
17	Hypotonia, development delay			Y	Nystagmus			Distal renal tubular acidosis	c.1786C>T p.Leu596Phe	c.879 + 1G>A p.Glu252_Glu293del	Itoh (2019) Brain 142(3):560–573
18	Tetraplegia, development delay, hypotonia		Pancytopaenia	Y	Nystagmus				c.1786C>T p.Leu596Phe	c.1786C>T p.Leu596Phe	Itoh (2019) Brain 142(3):560–573
19	Hypertonia, tetraplegia, dystonia, hypotonia		Anemia	Y					c.1786C>T p.Leu596Phe	c.1786C>T p.Leu596Phe	Itoh (2019) Brain 142(3):560–573
20	Hypotonia, severe development delay			Y	Nystagmus				c.1786C>T p.Leu596Phe	c.1786C>T p.Leu596Phe	Itoh (2019) Brain 142(3):560–573
21	Hypotonia, chorea, spasticity		Anemia, hypogammaglobulinemia	Y	Nystagmus				c.1786C>T p.Leu596Phe	c.566G>A p.Gly189Asp	Itoh (2019) Brain 142(3):560–573
22	Hypotonia, intellectual disability, slurred speech, ataxia, abnormal movements								c.1514G>A p.Arg505His	c.1597C>T p. Pro533Ser	Sun (2019) Neurol Genet.5(2):e565
23									c.1514G>A p.Arg505His	c.1597C>T p. Pro533Ser	Sun (2019) Neurol Genet.5(2):e565
24	Progressive neurocognitive decline, hypertonia, abnormal movements, ataxia			Y	Left eye blindness			Primary hypothyroidism	c.881T>C p.Ile294Thr	c.1760C>T p.Thr587Met	Sun (2019) Neurol Genet.5(2):e565
25	Developmente delay, development regression, hypertonia, hyperreflexia, progressive joint contractures, dysphagia	Y		Y	Vision loss, nystagmus			Renal tubular acidosis, mild monolateral hydronephrosis, failure to thrive	c.1281_1282insAGA p.Glu427_Leu428insAeg	c.1786C>T p.Leu596Phe	Sun (2019) Neurol Genet.5(2):e565
26	Developmente delay, hypotonia, intellectual disability, development regression, ataxia			Y					c.697C>G p.Leu233Val	c.697C>G p.Leu233Val	Sun (2019) Neurol Genet.5(2):e565
27	Apostural tetraparesis, severe development delay	Y	Pancitopenia, ring sideroblasts	Y	Leopard spot retinopathy, thin pRNF, retinal thinning, strabismus, poor fixation, delayed VEP		Pulmonary valve stenosis	Failure to thrive	c.815T>G p.Phe272Cys	c.1570T>C p.Cys524Arg	OUR PATIENT

*Y* yes, *y* year, *WM* white matter, *w* weeks, *d* days, *m* months

Our patient presents some new clinical findings never reported before. Presence of early-onset portal hypertension complicated by bleeding from esophageal varices is a new feature in *KARS1*-related syndromes, but it remains unclear how this occurs. In a previously reported child [6], hepatoportal sclerosis (HPS) was demonstrated on the liver biopsy. HPS is a rare case of non-cirrhotic portal hypertension characterized by sclerosis of the intrahepatic portal veins. HPS could present with normal or mildly abnormal liver function tests and, in advanced cases, with liver nodularity and atrophy producing an imaging appearance indistinguishable from that of cirrhosis [8]. It is tempting to speculate that energy failure due to low mitochondrial protein synthesis might be invoked in this case. Although the child described in the present report did not undergo a liver biopsy, her clinical presentation could be consistent with the diagnosis of HPS.

Our patient showed novel ocular findings. We observed early and severe signs of optic neuropathy and “leopard spot” retinopathy, a condition which has so far been associated with leukemia [9], uveal effusion syndrome [10], systemic argyrosis [11], pseudoxanthoma elasticum [12], Warburg syndrome [13], β thalassaemia [14] and neonatal adrenoleucodystrophy [15] but not with multisystem mitochondrial encephalopathies.

Whilst it was essential for the diagnosis of early optic neuropathy, SD-OCT scans failed to reveal alterations in choroidal and outer retinal layers corresponding to areas of retinal pigmentation, as described in adult *KARS1* patients [16].

Our patient also showed the simultaneous presence of cerebral and cerebellar atrophies combined with white matter calcifications seen at brain MRI imaging and the pancytopenia that are rare findings also pointing to a metabolic condition [5, 7].

From the analysis of the literature (Table 1) it appears that cerebral calcifications are present in 44.4% of patients (12/27) while other neurological characteristics are less common: cerebellar calcifications are seen in 5 of 27 patients, and cerebellar and cerebral atrophies in 4 and 2 children, respectively. The coexistence of these neurological features, as in our patient, is an extremely rare condition seldom seen in previous patients. An additional novel feature is the presence of pancytopenia in our patient. Mutations in *KARS1* can cause haematological alterations such as microcytic anemia, thrombocytopenia and hypogammaglobulinemia but pancytopenia occurred in a single child.

Due to the small number of patients, it is remains hard to draw genotype–phenotype correlations. An analysis of the literature (Table 1) shows that some features are shared by most of the patients, while others are rare and unrelated to type and site of mutation. It will be necessary to describe additional patients to improve our correlations.

In summary, our report expands the phenotype of new biallelic mutations in *KARS1* further stressing the importance to propose WES studies as first tier diagnostic method in infants with complex multisystem phenotypes. We also highlight the key role of SD-OCT examination in the multidisciplinary assessment of children with mitochondrial disorders.

## Supplementary information

**Additional file 1.** Methods.

**Additional file 2.** Full image of KARS Western blot.

**Additional file 3.** Sequencing of the region flanking the mutations using muscle cDNA analysis served to assess “semiquantitatively” the presence and the abundance of the wild-type and mutant transcripts.

**Additional file 4.** Full gel images of cDNA analysis.

## Data Availability

The datasets used and/or analysed during the current study are available from the corresponding author on reasonable request. The DNA sequencing data is available in NCBI BioProject under the accession number PRJNA673368. The direct web link to the NM_001130089.1 dataset and to the human reference hg19 genome that we used in this study are respectively https://www.ncbi.nlm.nih.gov/nuccore/NM_001130089.1 and https://www.ncbi.nlm.nih.gov/assembly/GCF_000001405.13.

## Abbreviations

MEH: Mitochondrial encephalohepatopathy
ARS: Aminoacyl tRNA synthetase
SD-OCT: Spectral domain optical coherence tomography
pRNFL: Peripapillary retinal nerve fiber layer
WES: Whole exome sequencing
HPS: Hepatoportal sclerosis
WB: Western Blot
