# Supplementary material for: Leopard-like retinopathy and severe early-onset portal hypertension expand the phenotype of KARS1-related syndrome: a case report
Source: BMC Med Genomics. 2021 Jan 21;14:25. doi: 10.1186/s12920-020-00863-1 (PMC7818779; doi:10.1186/s12920-020-00863-1)
Supplement: Supplementary file 1 — Additional file 1. Methods. [file 12920_2020_863_MOESM1_ESM.pdf]

DNA extraction

Written informed consent was obtained from the parents. Peripheral blood DNA was extracted using QIAamp Mini Kit (QIAGEN®, Hilden, Germany), according to manufacturer's instructions, and quantified by NanoDROP 2000 Spectrophotometer (Thermo Scientific, Waltham, MA, USA).

DNA library preparation and Sequencing

To construct DNA libraries we used a strategy based on enzymatic fragmentation to produce dsDNA fragments followed by End repair, A-tailing, adapter ligation and library amplification (Kapa Biosystems, Wilmington, MA). Libraries were hybridized with the protocol SeqCap EZ Exome v3 (Nimblegen, Roche, Basel, Switzerland) and sequenced by NextSeq500 (Illumina Inc., San Diego, CA).

Assembly, Variant Calling

The reads were aligned with the human reference hg19 genome using Burrows-Wheeler Aligner (BWA) (1), mapped and analyzed with the IGV software (Integrative Genome Viewer, 2013 Broad Institute) (2). Downstream alignment processing (i.e. alignment sorting, indexing, deduplication and base quality score recalibration) was performed with the Genome Analysis Toolkit Unified Genotyper Module (GATK) (3) SAMtools (4) and Picard Tools (http://picard.sourceforge.net/). The GATK was used to obtain a set of raw single nucleotide variant (SNV) calls, which were filtered using the following criteria: QD < 5.0, DP < 5, FS > 60.0, QUAL < 30.0. Variants were annotated using Annovar tool (5) to obtain information such as variant frequency in different populations and the predictions of the variant effect using different methods (SIFT, Polyphen2, MutationTaster, MutationAssessor, FATHMM and FATHMM MKL). A variant effect consensus was obtained by categorizing the variant effects obtained for each method in deleterious and non-deleterious, then counting the number of deleterious effects to prioritize variants. WES trios- analysis was performed to established the inheritance pattern. Variants were classified and scored in agreement with the interpretation guidelines of the American College of Medical genetics and Genomics (ACMG) (6). In details, we selected only non-synonymous, short insertion/deletion or splice-site variants (30 bp splice acceptor, 30 bp splice donor) with the following characteristics:

- variants not present or with a minor allele frequency ≤ 0.01 for autosomal recessive (AR) and with a minor allele frequency ≤ 0.001 for autosomal dominant (AD)-transmitted genes in population database "1000 Genomes Project", "Exome Variant Server" (dbSNP147), ExAC, gnomAD.

-variants predicted as damaging by at least 4 over 6 in silico tools (Polyphen-2, SIFT, Mutation Taster, FATHMM, FATHMM MKL, Mutation Assessor).

- variants were researched in a database that reported disease-causing mutations, we used ClinVar and the Human Gene Mutation Database (HGMD) Professional (http://www.hgmd.cf.ac.uk/ac/index.php) updated to 2019.

- variants correctly segregating within the family or representing de novo variants.

- different cut-off values for the ratio of novel allele to reference allele reads were used. For analyses of homozygous variants, we included variants with at least 80 % novel allele reads. Heterozygous variants were included only when called in at least 10 % and at most 80 % of all reads. Considering missense variants, we only included variants with a CADD-value of at least 10 (other variants are considered benign). The remaining variants were inspected with the Integrative Genomics Viewer to exclude sequencing errors in repetitive regions. Variants of all individuals were analyzed for known genes compiled from OMIM, regarding the inheritance model and the phenotype of each individual.

Variant validation

Prioritized variants were validated by Sanger sequencing. Splice site variants have been evaluated using in silico prediction (BGDP http://www.fruitfly.org/ and HSF http://www.umd.be/HSF/HSF.shtml).

References

1. Li H, Durbin R: Fast and accurate short read alignment with Burrows-Wheeler transform BWA. *Bioinformatics* 25: 1754-1760, 2009
2. Thorvaldsdóttir H, Robinson JT, Mesirov JP: Integrative Genomics Viewer (IGV): high-performance genomics data visualization and exploration IGV. *Brief Bioinform* 14:178-192, 2013
3. McKenna A, Hanna M, Banks E, Sivachenko A, Cibulskis K, Kernytsky A, Garimella K, Altshuler D, Gabriel S, Daly M, DePristo MA: Next-generation DNA sequencing data. *Genome Res* 20: 1297-1303, 2010
4. Li H, Handsaker B, Wysoker A, Fennell T, Ruan J, Homer N, Marth G, Abecasis G, Durbin R; 1000 Genome Project Data Processing Subgroup: The sequence alignment/map (SAM) format and SAMtools. *Bioinformatics* 25: 2078-2079, 2009
5. Wang K, Li M, Hakonarson H: ANNOVAR: functional annotation of genetic variants from high-throughput sequencing data. *Nucleic Acids Res* 38: e164. 2010
6. Richards S, Aziz N, Bale S, Bick D, Das S, Gastier-Foster J, Grody WW, Hegde M, Lyon E, Spector E, Voelkerding K, Rehm HL: ACMG Laboratory Quality Assurance Committee: Standards and guidelines for the interpretation of sequence variants: a joint consensus recommendation of the American College of Medical Genetics and Genomics and the Association for Molecular Pathology. *Genet Med* 17: 405-424, 2015
