# Supplementary figures and images for: Leopard-like retinopathy and severe early-onset portal hypertension expand the phenotype of KARS1-related syndrome: a case report
Source: BMC Med Genomics. 2021 Jan 21;14:25. doi: 10.1186/s12920-020-00863-1 (PMC7818779; doi:10.1186/s12920-020-00863-1)

PROTEIN LADDER Ctrl\_1 Pt Ctrl\_2

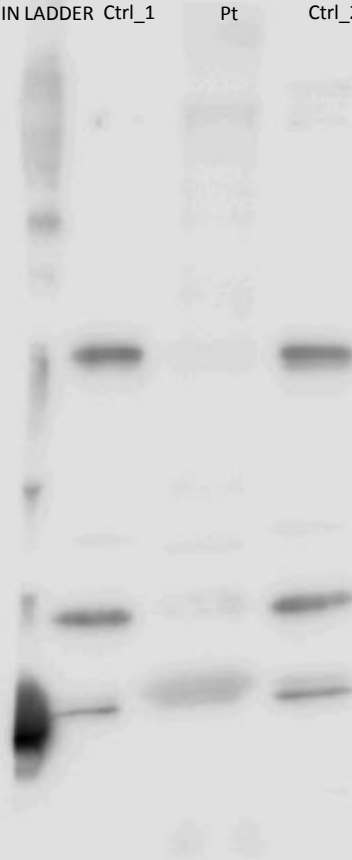

Supplement: Supplementary file 2 — Additional file 2. Full image of KARS Western blot. [file 12920_2020_863_MOESM2_ESM.pdf]

## Slide 1
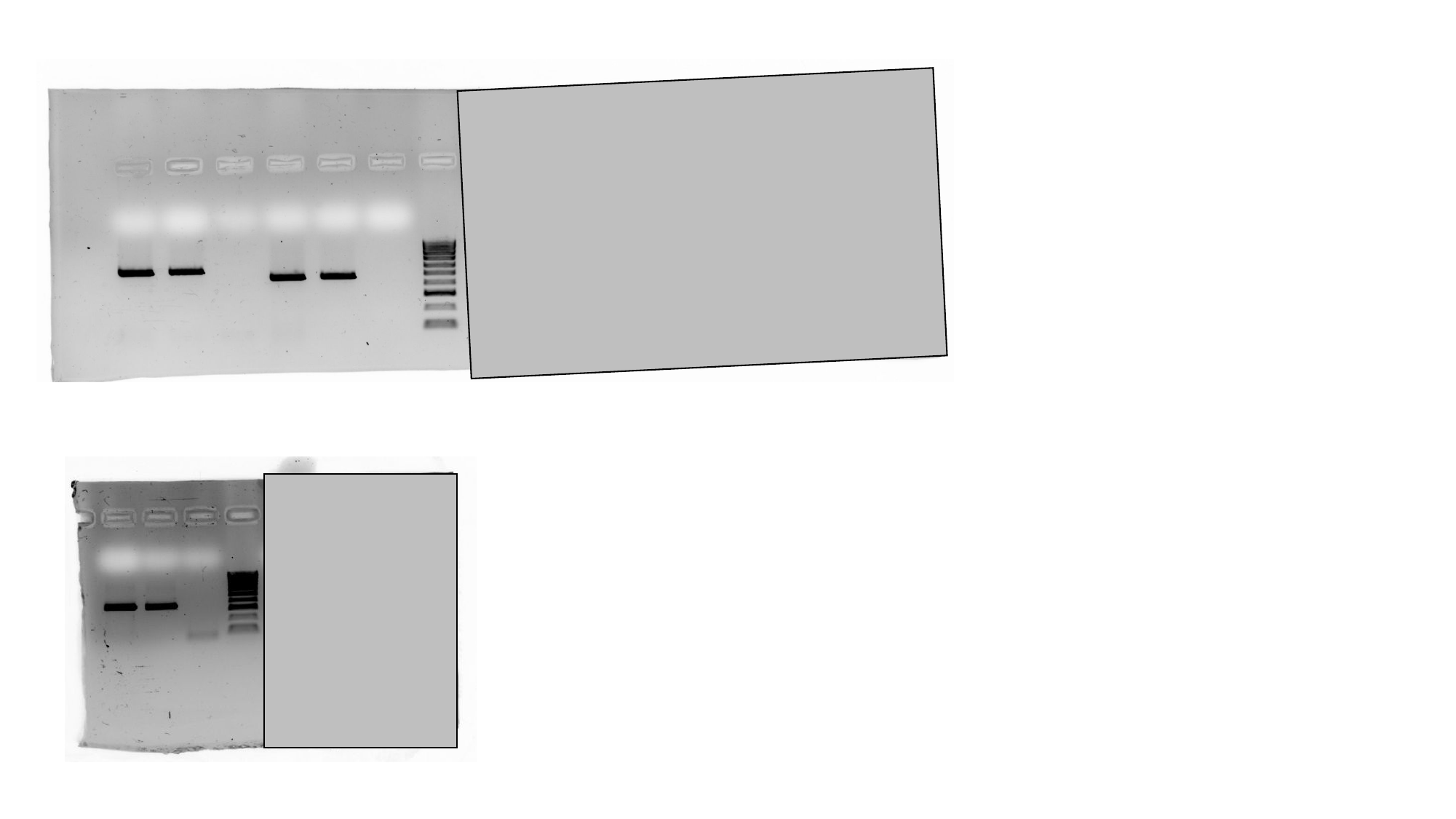

Supplement: Supplementary file 4 — Additional file 4. Full gel images of cDNA analysis. [file 12920_2020_863_MOESM4_ESM.pptx]
