# Supplementary material for: Leopard-like retinopathy and severe early-onset portal hypertension expand the phenotype of KARS1-related syndrome: a case report
Source: BMC Med Genomics. 2021 Jan 21;14:25. doi: 10.1186/s12920-020-00863-1 (PMC7818779; doi:10.1186/s12920-020-00863-1)
Supplement: Supplementary file 3 — Additional file 3. Sequencing of the region flanking the mutations using muscle cDNA analysis served to assess “semiquantitatively” the presence and the abundance of the wild-type and mutant transcripts. [file 12920_2020_863_MOESM3_ESM.ppt]

## Slide 1
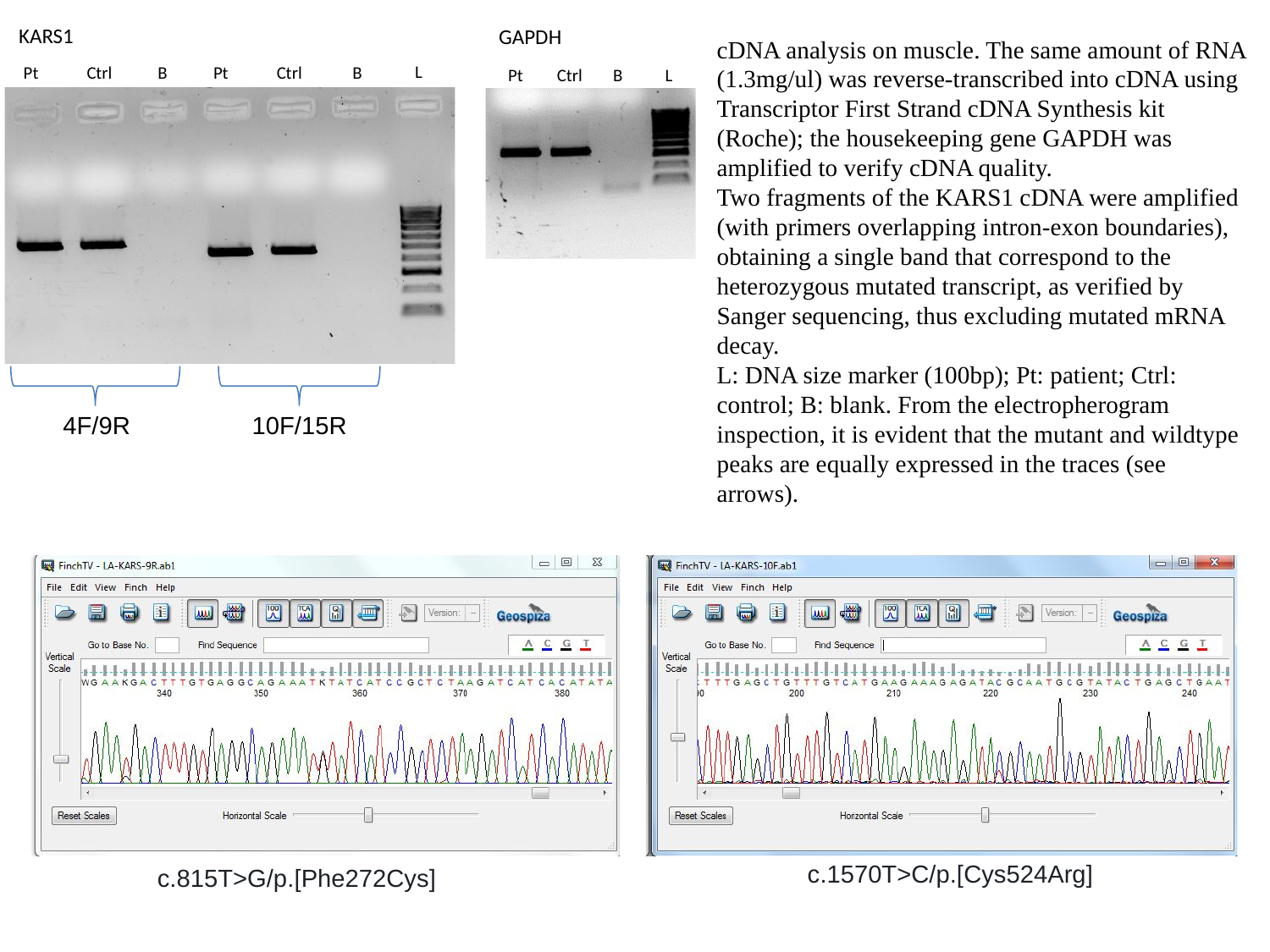

cDNA analysis on muscle. The same amount of RNA (1.3mg/ul) was reverse-transcribed into cDNA using Transcriptor First Strand cDNA Synthesis kit (Roche); the housekeeping gene GAPDH was amplified to verify cDNA quality.
Two fragments of the KARS1 cDNA were amplified (with primers overlapping intron-exon boundaries), obtaining a single band that correspond to the heterozygous mutated transcript, as verified by Sanger sequencing, thus excluding mutated mRNA decay.
L: DNA size marker (100bp); Pt: patient; Ctrl: control; B: blank. From the electropherogram inspection, it is evident that the mutant and wildtype peaks are equally expressed in the traces (see arrows).
KARS1
GAPDH
L
Pt
B
Ctrl
Pt
B
Ctrl
Pt
B
L
Ctrl
4F/9R
10F/15R
c.1570T>C/p.[Cys524Arg]
c.815T>G/p.[Phe272Cys]
